# Present state of intestinal transplantation in Japan

**DOI:** 10.1007/s00383-023-05552-5

**Published:** 2023-09-27

**Authors:** Takehisa Ueno, Motoshi Wada, Eri Ogawa, Toshiharu Matsuura, Yohei Yamada, Seisuke Sakamoto, Hiroomi Okuyama

**Affiliations:** 1 Department of Pediatric Surgery, Osaka University of Graduation School of Medicine, 2-2 Yamadaoka, Suita, Osaka 565-0871 Japan; 2 Department of Pediatric Surgery, Tohoku University Graduate School of Medicine, 1-1 Seiryo-cho Aoba-ku, Sendai-shi, Miyagi-ken Japan; 3 Department of Surgery, Kyoto University Graduate School of Medicine, Yoshida-Konoe-cho, Sakyo-ku, Kyoto, 606-8501 Japan; 4 Department of Pediatric Surgery, Graduate School of Medical Sciences, Kyushu University, 744 Motooka, Nishi-ku, Fukuoka, Japan; 5 Department of Pediatric Surgery/Transplant Center, Keio University School of Medicine, 35 Shinanomachi, Shinjuku, Tokyo 160-8582 Japan; 6 Organ Transplantation Center, National Center for Child Health and Development, 2-10-1 Okura, Setagaya-ku, Tokyo 157-8535 Japan

**Keywords:** Intestinal failure, Short bowel syndrome, Motility disorder, Parental nutrition, Small bowel transplantation, Everolimus

## Abstract

**Introduction:**

Intestinal transplantation (ITx) is the ultimate treatment for intestinal failure (IF). In Japan, most cases of IF are a result of pediatric disease, including secondary or congenital intestinal disease or allied disorders of Hirschsprung’s disease. Here, we report the results of the Japanese ITx registry.

**Methods:**

A web-based survey form was completed. We investigated the number, age, sex, indication, surgical procedure, immunosuppressants, postoperative course, and the effects of transplantation in patients who underwent cadaveric or living-donor ITx.

**Results:**

By the end of 2022, 42 cases of ITx have been performed in 38 patients in Japan. The donor sources included cadavers (29 cases) and living donors (13 cases). The surgical method was isolated ITx (*N* = 40) and combined liver and ITx (*n* = 2). Survival rates were 92%, 73%, and 59% at 1 year, 5 years, and 10 years, respectively. Ninety percent of patients completely discontinued parenteral nutrition. Approximately 80% of the patients had a performance status of 1 or less, indicating that the QOL of patients after ITx was extremely good.

**Conclusion:**

The results of ITx are acceptable to treat IF patients and the QOL after transplantation is also good.

## Introduction

Intestinal failure (IF) is the inability of the small bowel to absorb nutrition for digestion and absorption of adequate nutrients and fluids for survival and growth, commonly as a result of short bowel syndrome (SBS) or motility disorders (MD) [1]. In Japan, most cases of IF are a result of pediatric disease including SBS secondary to congenital intestinal disease or MD such as allied disorders of Hirschsprung’s disease. Patients with IF are usually dependent on parenteral nutrition (PN). PN is an effective treatment and home PN is the standard of care for patients with IF [2]. The prognosis for IF has improved dramatically owing to the development of PN. However, prolonged PN causes numerous complications, including central venous catheter infection, thrombosis of venous access, and IF-associated liver disease (IFALD). Some children with IF develop life-threatening complications despite receiving the best medical and surgical treatment [3].

Intestinal transplant (ITx) is the ultimate treatment for IF and is already covered by national health insurance in Japan. ITx can significantly improve a patient’s prognosis and improve their quality of life (QOL). ITx remains an acceptable treatment for patients with IF, but it is generally reserved for patients who develop severe and life-threatening complications despite medical or surgical treatment, or those who are not able to maintain a satisfactory QOL. Therefore, we investigated the current state of ITx in Japan and assessed it for future improvement. The purpose of this paper is to describe our findings related to the current state based on the Japanese ITx registry.

## Methods

### Data collection

Patient data were collected in the Japanese intestinal transplant registry (ITR), which was initiated in 2006. This survey and publication were approved by the Osaka University Hospital Ethics Committee (IRB) (Approval Number: 12233-5). All centers performing ITx in Japan are invited to contribute to this database. A survey request was sent to each ITx facility, and each institution completed a case survey form on the website of the data center. All data were entered into the REDCap database (Nashville, TN, USA) [4]. Cumulative reports were made from these data. Data collection includes recipient demographics, pretransplant diagnosis and status, type of transplant, age, sex, cadaveric or living donor, and post-transplant status—assessed once yearly—including immunosuppressive regimen and complications. Patient follow-up was conducted at least once per year and was collected at the end of each year.

### Analysis

The first case of ITx in Japan was performed in 1996. From the first case, data were accumulated to the end of 2022, and data collected between 1996 and 2022 were analyzed. Descriptive statistics were used to summarize patient demographics, procedures, underlying disease, immunosuppression, patient and graft survival, outcome of ITx, and QOL. Graft survival was defined as the time from transplant until graft failure or recipient death at 1 year, 5 years, and 10 years post-transplant. Patient and graft survival were assessed based on patient age at transplant, indication for transplant (SBS vs MD), primary or retransplant, donor type (living vs deceased), type of induction therapy, and liver transplant (yes vs no). Graft function was analyzed with PN dependency and intravenous fluid requirement. QOL was evaluated with performance status (PS) [5]. PS grades were as follows. Grade 0: Fully active; no performance restrictions. Grade 1: Strenuous physical activity restricted; fully ambulatory and able to carry out light work. Grade2: Capable of all self-care but unable to carry out any work activities. Up and about > 50% of waking hours. Grade 3: Capable of only limited self-care; confined to bed or chair > 50% of waking hours. Grade 4: Completely disabled; cannot carry out any self-care; totally confined to bed or chair. Graft function and QOL was evaluated based on data from surviving recipients with functioning grafts for longer than 1 year.

### Statistical analysis

The survival rate was determined using the Kaplan–Meier method, and the log-rank method was used for the test. Data were analyzed and Kaplan–Meier plots were generated using the JMP software package, version 8.0 (SAS Institute) and *p* < 0.05 was considered statistically significant.

## Results

### Demographic characteristics of the study patients

**Fig. 1:**
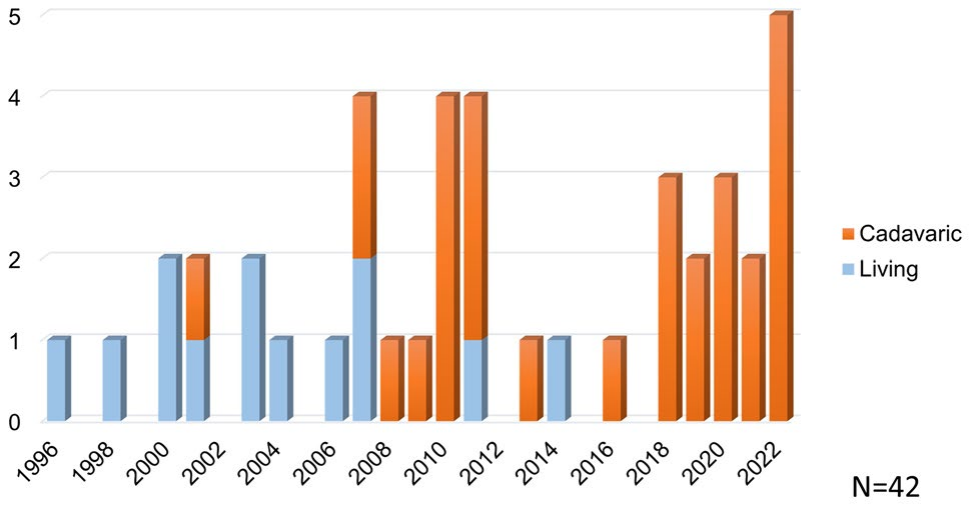
Annual case number of intestinal transplant

**Fig. 2:**
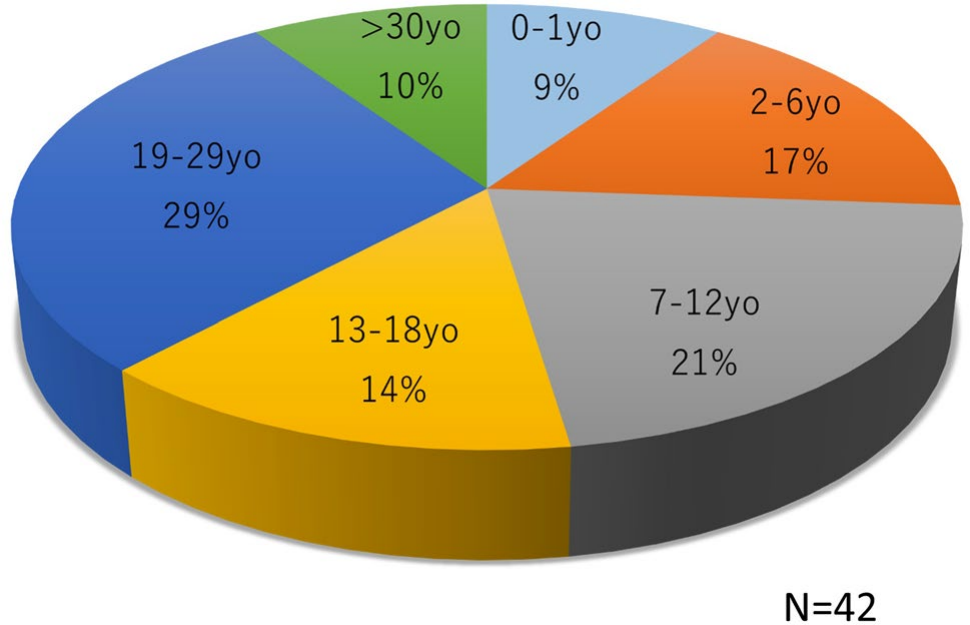
Age of recipient. *yo* year-old

**Fig. 3:**
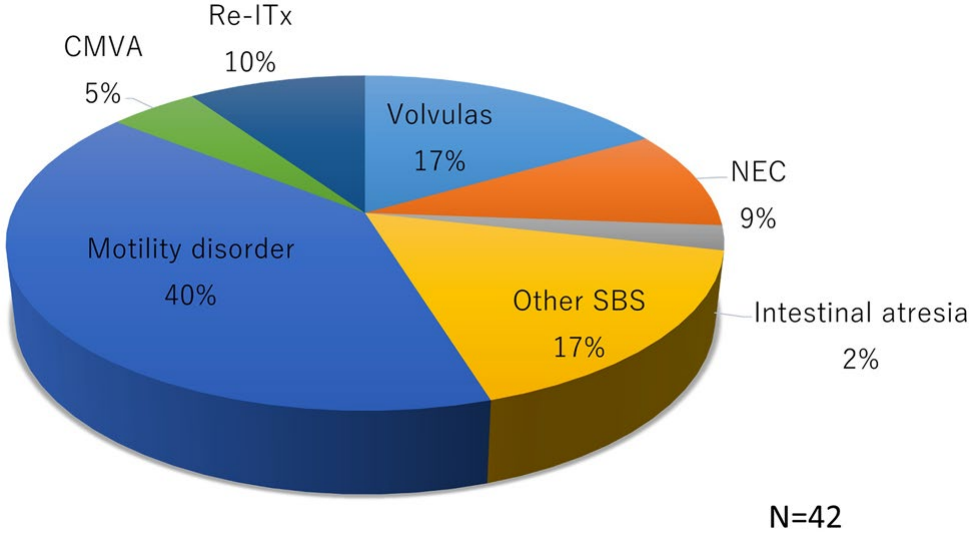
Indications for intestinal transplant. Motility disorder includes hypogangliosis (*n* = 10), chronic idiopathic intestinal pseudoobstruction (*n* = 4), immaturity of ganglia (*n* = 1) and others (*n* = 2). *CMVA* congenital microvillous atrophy, *NEC* necrotizing enterocolitis, *Re-ITx* re-intestinal transplant, *SBS* short bowel syndrome

All ITx cases in Japan were registered and analyzed. By the end of December 2022, 42 ITxs had been performed for 38 patients. The donor sources included cadavers (29 cases) and living donors (13 cases). Figure 1 shows the annual number of cadaveric and living-donor ITxs. Twenty-four of the ITxs occurred in males and 18 in females. Figure 2 shows the age at transplant. Although most cases of ITx in Japan are based on childhood diseases, nearly 40% of cases are in adults aged 19 years or older. Figure 3 shows the indication for ITx. MD includes hypogangliosis (*n* = 10), chronic idiopathic intestinal pseudoobstruction (*n* = 4), immaturity of ganglia (*n* = 1) and others (*n* = 2). In recent years, SBS due to massive resection of the small bowel has increased, and the number has reached the same as MD. In addition, with the increase in the number of ITx, retransplant due to post-transplant graft failure has also increased.

### Procedure types

The surgical procedures were isolated transplantation (*n* = 42) and simultaneous liver and intestinal transplantation (*n* = 2). One was from a cadaveric donor and the other was from two living donors. Among the isolated ITxs, seven cases had a living-donor liver transplant before or after ITx due to IFALD. Since ABO blood type matching is desirable for ITx, 37 cases of donors matched the ABO blood type and 5 cases were compatible. Blood type–incompatible transplants have not been performed. The varying lengths of the small intestine used as the graft was less than 150 cm (*n* = 7), 150–199 cm (*n* = 10), 200–249 cm (*n* = 9), 250–299 cm (*n* = 7), and longer than 300 cm (*n* = 8). The use of grafts longer than 150 cm has become mainstream related to the increase in cadaveric donors. Forty-five percent of grafts included the ileocecum valve with the colon. Regarding the reconstruction blood supply, 49% of the venous reconstruction was inferior vena cava return and 51% was portal vein return including the superior mesenteric vein. The artery reconstruction used the aorta (*n* = 28), superior mesenteric artery (*n* = 11), and others (*n* = 3).

### Patient and graft survival

**Fig. 4:**
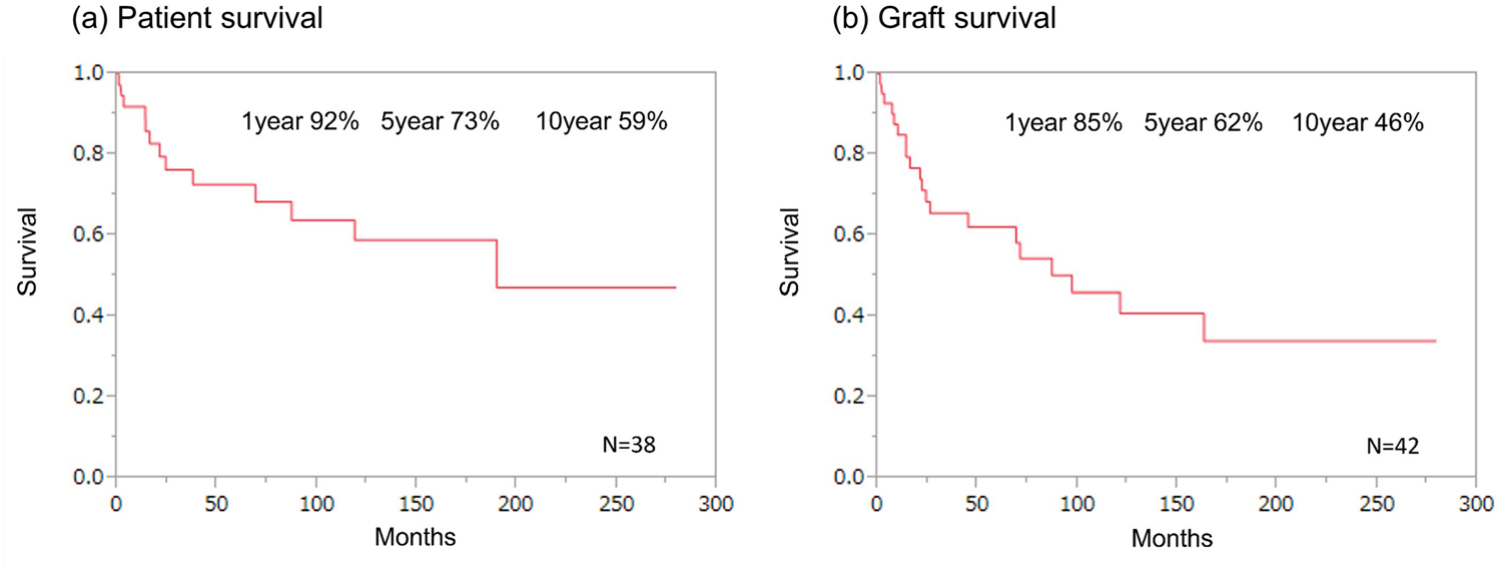
Overall survival **a** Overall patient survival. **b** Overall patient survival

Cumulative patient survival until December 2022 is shown in Fig. 4a. Patients had a 1-year survival rate of 91%, a 5-year survival rate of 73%, and a 10-year survival rate of 59%. Figure 4b shows similar graft survival rates of 86%, 64%, and 47% at 1 year, 5 years, and 10 years after ITx, respectively. Patients and graft survival have been improved last 2 decades, however, the rate of improving was plateau recently (Data not shown).

**Fig. 5:**
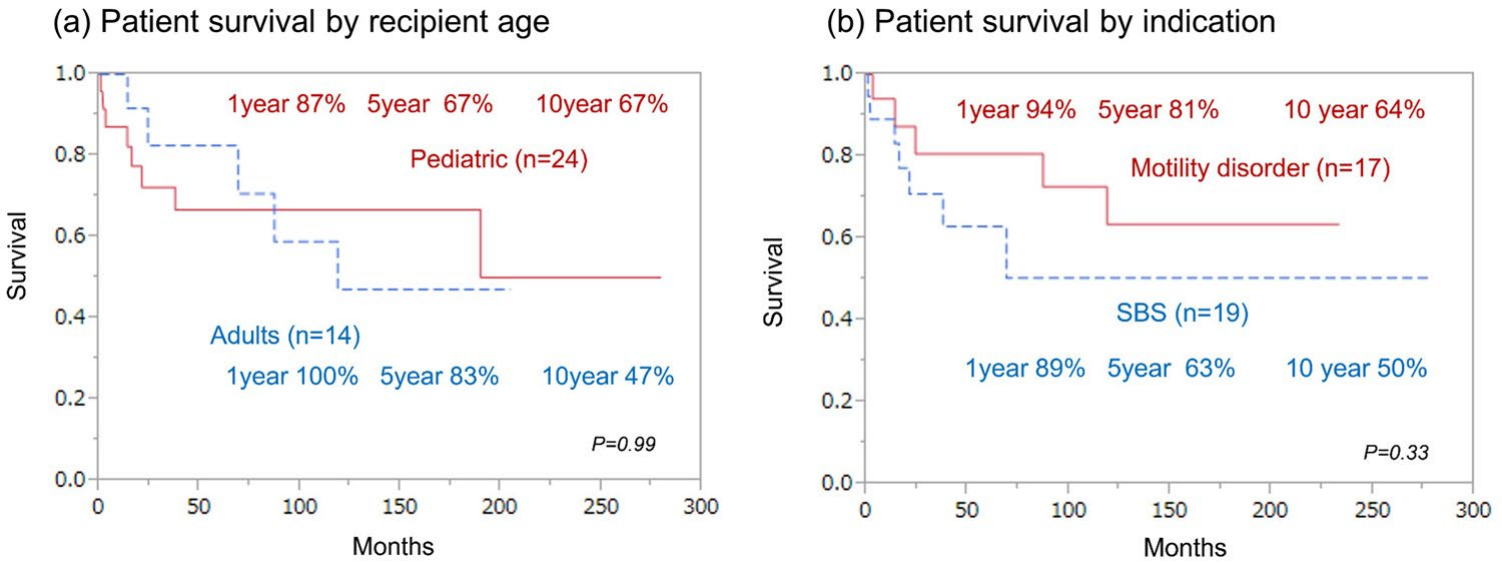
Patient survival **a** By recipient age (Pediatric vs Adult): The solid line indicates pediatric survival. The dashed line indicates adult survival. Pediatric means under 19 year olds. Adult means 19 year olds and over. **b** By indication (SBS vs Motility disorder): The solid line indicates motility disorder survival. The dashed line indicates SBS survival. SBS means short bowel syndrome

Pediatric patients have a 1-year survival rate of 87%, a 5-year survival rate of 67%, and a 10-year survival rate of 67%. Whereas adult patients have a 1-year survival rate of 100%, they have a 5-year survival rate of 83% and a 10-year survival rate of 47% (*p* = 0.99). No statistically significant difference was observed (Fig. 5a).

Patients with SBS have a 1-year survival rate of 89%, a 5-year survival rate of 63%, and a 10-year survival rate of 50%. Comparatively, patients with MD have a 1-year survival rate of 94%, a 5-year survival rate of 81%, and a 10-year survival rate of 64% (*p* = 0.33). No statistically significant difference was observed. (Fig. 5b).

Graft survival after primary transplantation was 86%, 64%, and 51% at 1 year, 5 years, and 10 years, respectively. Graft survival in re-transplantation was 75% and 38% at 1 year and 5 years, respectively. Graft survival of isolated ITx was 88%, 64%, and 49% at 1 year, 5 years, and 10 years, respectively. Graft survival in combined/sequential liver and intestine transplant was 70%, 53%, and 26% at 1 year, 5 years, and 10 years, respectively (*p* = 0.29).

Thirteen patients died after ITx. The reasons for death included infection (*n* = 4), liver failure (*n* = 3), CMV pneumonia, post-transplant lymphoproliferative disorder, Epstein–Barr virus-associated hemophagocytic syndrome, brain abscess, intraperitoneal bleeding, and persistent pancreatitis (*n* = 1 each). Infections are still important issues in the postoperative management of ITx. However, it is unclear whether the cause of the infection was the infection itself or the result of rejection; thus, this is necessary to clarify in the future.

### Living donor

**Fig. 6:**
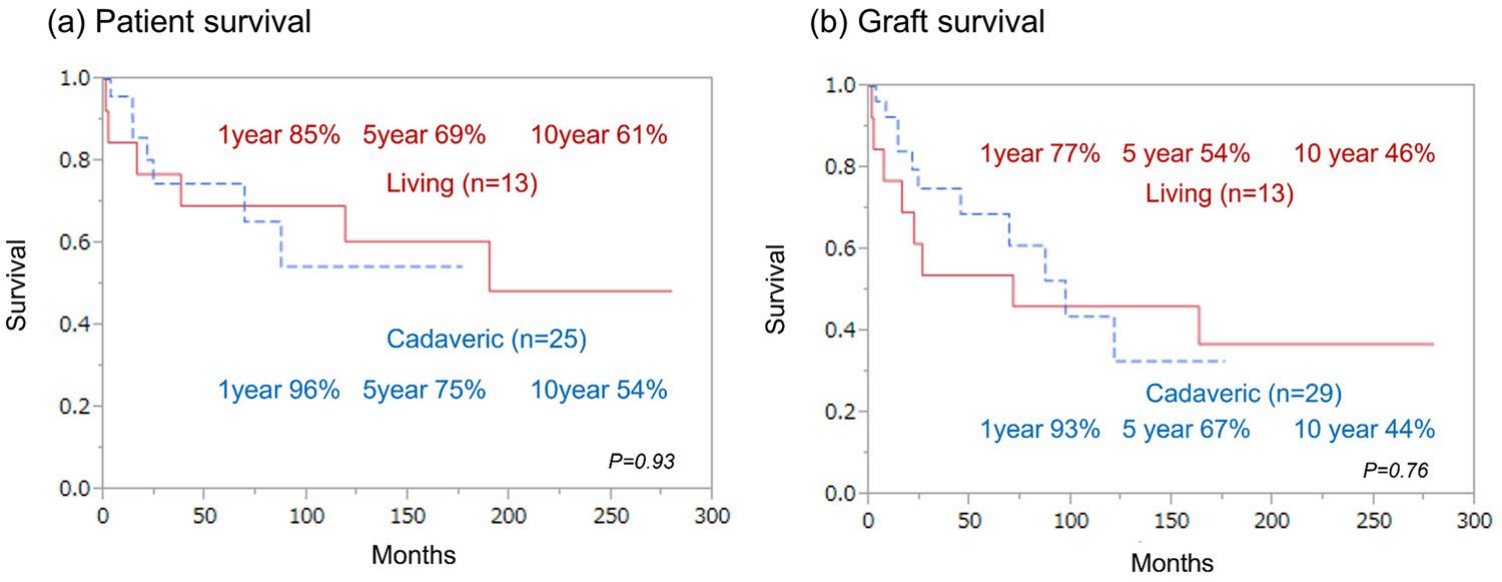
Survival by donor type (Cadaveric vs Living) **a** Patient survival by donor type. **b** Graft survival by donor type. The solid line indicates living-donor survival. The dashed line indicates cadaveric donor survival

Figure 6a, b compares the patient survival and graft survival for cadaveric and living donors. Although no significant difference was observed between cadaveric donors and living donors, considering that living-donor ITxs were performed a relatively long ago, there is a possibility that the timing of the transplantation is biased.

### Immunosuppression

**Fig. 7:**
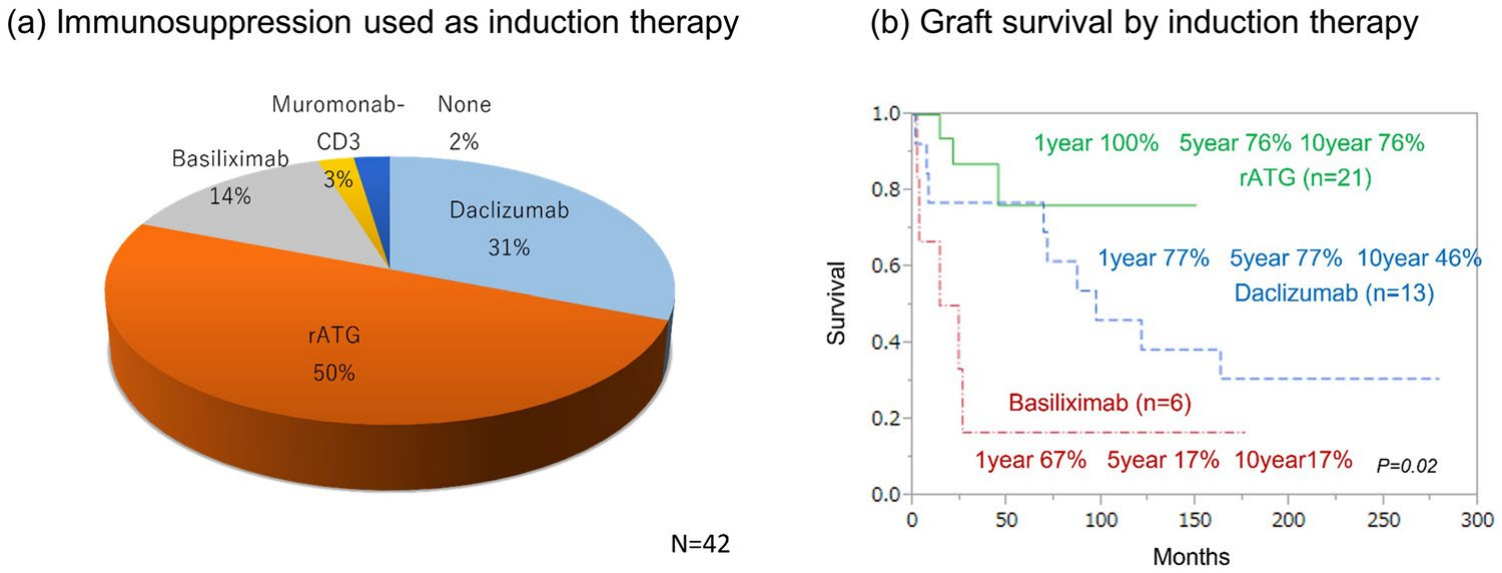
Induction immunosuppression **a** immunosuppression used as induction therapy. **b** Graft survival by induction therapy. The solid line indicates rATG survival. The dashed line indicates Daclizumab survival. The dash-dotted line indicates Basiliximab survival. *rATG* rabbit anti-thymus globulin

Immunosuppression, mainly with tacrolimus, is used in all cases. In addition, induction immunosuppressive therapy (Induction) is used because ITx is prone to rejection. The drugs used are shown in Fig. 7a. Previously, Daclizumab (Zenapax®) was used for induction; recently, however, rATG (Thymoglobulin®) has become the most common drug for induction, because Daclizumab was not available on the market. Figure 7b compares the graft survival rate according to the type of induction immunotherapy. rATG maintains the same results as Daclizumab, and induction therapy using rATG is considered appropriate. All cases continued with tacrolimus for maintenance immunosuppression. Everolimus was added for four patients on maintenance tacrolimus.

### Graft function and QOL

**Fig. 8:**
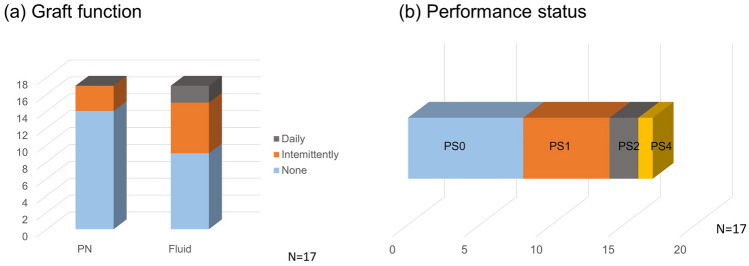
Post-transplant status one year after intestinal transplant **a** Graft function (PN and fluid dependency): *PN* parenteral nutrition. Fluid means intravenous fluid requirement. **b** Performance status after intestinal transplant. *PS* performance status

For the outcome of ITx, both an improvement in lifetime prognosis and improvement in QOL are important; these effects of ITx among 17 patients with functional grafts more than 1 year after ITx were assessed. QOL was evaluated by PN dependence, iv (intravenous) fluid withdrawal, stoma closure, and PS in patients with a functional graft more than 1 year after ITx. Figure 8a shows graft function. Approximately, 60% of patients achieved stoma closure, after which stoma care was no longer required. All were partially discontinued from PN and approximately 90% were able to wean completely from PN. Only approximately 30% of patients required iv fluid replacement. Figure 8b shows the QOL of ITx patients evaluated by PS. Approximately 80% of patients have a PS of 1 or less, indicating that the QOL of patients after ITx is extremely good.

## Discussion

Global data analyses were reported previously based on international ITR. This has shown growth and improvement in graft survival rates over the last 2 decades [6, 7]. ITx remains the ultimate treatment for patients with irreversible IF who develop life-threatening complications associated with PN. However, the number of ITxs remains very low in East Asia [8]. Similarly, there are only a few cases per year performed in Japan. In our experience, patients have a 1-year survival of 91%, a 5-year survival rate of 73%, and a 10-year survival rate of 59%. Our results in Japan are comparable with results worldwide and are considered acceptable results for the treatment of IF.

According to a 2011 survey conducted by the Ministry of Health, Labour and Welfare Science Research Fund in Japan [9], it is estimated that there are nearly 200 potential patients waiting for ITx nationwide. There were two major factors contributing to the relative rarity of ITx in Japan. One is the lack of available organs. Historically, few organs from deceased donors were obtainable in Japan. As with other solid organs, most intestinal transplants in Japan were previously completed with living and related donors. However, this situation has changed due to a new organ transplantation law, effective July 2010.

The other factor was a shortage of pediatric donations. Although most cases of ITx in Japan are based on childhood diseases, nearly 40% of completed cases are adults aged 19 years or older. Previously, the laws on organ transplantation banned donors younger than 15 years of age. This is the main reason why only one-third of recipients were younger than 7 years. ITx for infants was not previously possible because of organ size mismatch. Such patients will benefit from intestinal transplants in the future.

Some patients developed IFALD with SBS. Detection of IFALD is important [10]; however, some of these patients experience liver failure. These patients require simultaneous liver–intestine transplants. A combined liver–intestine transplant has less risk of acute rejection than an isolated ITx, because the liver may have protective effects on the intestine [11]. There may be a benefit from the addition of the liver component attributable to a reduction in DSA and antibody-mediated rejection [12]. The current organ allocation system does not allow for easy simultaneous combined liver–intestine transplants, because liver is allocated to the high status liver waiting patients like fulminant hepatitis due to shortage of donation in Japan. Some isolated ITxs from deceased donors in advance or following living-donor liver transplant—sequential liver–intestine transplant—have been attempted. Among isolated ITx cases, seven cases had living-donor liver transplants before or after ITx due to IFALD. These cases were intended to be simultaneous liver–intestinal transplants and multivisceral transplantation is a good choice for such patients [13]. However, the allocation system does not accommodate this option. In the future, it will be necessary to establish a system that allows multi-organ transplantation for patients with liver failure in Japan. In addition, grafts including ileocecal valves are thought to have increased because of the increase in cadaveric donations and the favorable results of grafts with ileocecal valves [14].

The intestine has long been seen as a “forbidden” organ for transplantation [15]. With the development of better immunosuppressive management and improvements in our understanding, the survival rate of ITx has improved [16], although issues with chronic rejection persist [17]. Renal function is a major challenge after ITx [18, 19]. As well, sirolimus has the potential to improve the side effects of calcineurin inhibitors [20]. The use of sirolimus in immunosuppression is thought to be favorable [21]. Despite this, everolimus is used as a third-line agent instead of sirolimus, because rapamycin had not been available on the Japanese market [22].

Limitations are present in this type of retrospective analysis because of its nature as a registry report. The data are limited by the information reported and may have some missing or incomplete data, although data clearance was undertaken to maximize these errors.

In conclusion, the outcome of ITx is acceptable to treat IF patients, and the QOL after transplantation is also good. A future objective is to provide patients receiving ITx with excellent long-term graft function.
